# Editorial: Aging and the microbiome

**DOI:** 10.3389/fragi.2024.1522703

**Published:** 2024-11-15

**Authors:** Claudia Villicaña, Chiara H. Moretti, Franck Carbonero

**Affiliations:** ^1^ CONAHCYT-Laboratorio de Biología Molecular y Genómica Funcional, Centro de Investigación en Alimentación y Desarrollo A.C., Culiacán, Sinaloa, México; ^2^ Department of Molecular and Clinical Medicine, Sahlgrenska Academy, Gothenburg University, Gothenburg, Sweden; ^3^ Department of Nutrition and Exercise Physiology, Elson S. Floyd College of Medicine, Washington State University Health Sciences Spokane, Spokane, WA, United States

**Keywords:** microbiome, biomarkers, gut, skin, oral

Aging is a cellular process that naturally occurs throughout lifespan and involves the accumulation of deleterious effects, triggering progressive physiological and intellectual deterioration. However, phenotypes associated with aging appear differently in time and extent in the population, and often, they are linked to the development of certain diseases. Several determinants such as genetic, environmental and lifestyle factors have been demonstrated to influence the “biological age” becoming critical for intervention and preventing age-related diseases contributing to healthy aging. For several years, the microbiome has been linked to the aging process as a relevant component that regulates several physiological processes. Most of our microbiome knowledge comes from gut studies, thus the characterization of microbial communities located at other organs and tissues will expand our understanding of microbial-host interaction, giving us the opportunity to discover novel biomarkers and targets for developing interventional strategies to diagnose and delay age-associated diseases.

In this Research Topic, Claudia Villicaña, Chiara H. Moretti and Franck Carbonero focused on the roles of microbiome in the aging process and its association with aging-associated diseases. This Research Topic includes a collection of three original research articles and one review article that provide interesting findings revealing novel factors influencing microbiome composition, microbiome shifts in facial skin, saliva and gut, the role of inflammation, modulation of gene expression associated with critical cell processes in aging tissues and the relationship between molecular biomarkers and older adults with cancer, providing novel insights for the diagnoses and treatment of aging-related diseases.

Several studies have shown that alterations in gut microbiome composition occur during aging, often accompanied by inflammation, although the precise mechanisms impairing intestinal dysfunction have been poorly addressed. Using a mouse model at different ages, Forsyth et al. evaluated the changes in the microbiome in feces and found a correlation with the gene expression profile of colonic crypts obtained through RNA-seq. Fecal analysis revealed differences in the microbial community structure between 2-month-old mice and 15- and 25-month-old mice displaying a significant increase in dysbiotic Gram-negative bacteria over time. Moreover, the association of microbiome modulation and differential gene expression in colonic crypt epithelial cells suggested that several genes involved in critical cellular processes such as senescence, antimicrobial peptides, cell junctions, inflammation and intestinal barrier were altered in the course of aging, providing novel insights into the mechanistic role of the microbiome as a hallmark of aging and cell dysfunction.

In the context of chronic diseases, patients may experience a functional decline or worsening disability, which is typically associated with increased mortality in older adults. Functional independence is prioritized over prolonged survival in older adults with chronic diseases, becoming critical to find molecular markers to predict treatment toxicities to identify vulnerable groups, which, in conjunction with assessing geriatric parameters, may improve quality of life (QoL), independent living and reduced financial healthcare costs. Lung cancer is one of the primary diseases in older adults, but prognosis in this age group is uncertain because most clinical trials are carried out in younger adults, complicating the prediction of treatment toxicity, disease response and functional status. In this regard, Grogan et al. performed a prospective study on adults over 60 years old diagnosed with non-small-cell lung cancer. In this study, the patients were assessed on physical and cognitive evaluations, QoL, treatment toxicity, blood marker analysis, and microbiome characterization. Interestingly, analyses revealed a higher independence score by instrumental activities of daily living (IADLs), QoL and short physical performance battery (SPBB) and positively correlated with specific components of microbiome such as *Candidatus Gastranaerophilales*, *Lactobacillus rogosae* and *Enterobacteria* phage P4. In contrast, the presence of *Romboutsia ilealis*, *Streptococcus* and *Lachnoclostridium*, were associated with worsened conditions of physical independence. These findings suggest that microbiome can be used as molecular markers to predict good outcomes in older patients, or being targets for designing tailored care interventions to improve microbiome health and prevent functional disability.

Despite most microbiome studies have been done in the gut, recent studies have shown that microbiomes from different tissues and organs have different compositions, unveiling additional factors in addition to aging and disease that also influence microbiome profiles. In this context, Pagac et al. provided evidence of the hormone-driven impact on the microbiome composition of human facial skin in pre- and post-menopausal women. Global microbiome analysis revealed that the *Cutibacterium* genus was the most abunant on skin, but significantly more abundant in the pre-menopausal group, meanwhile *Streptococcus* was significantly increased in post-menopausal group. Moreover, microbiome diversity showed a higher diversity in post-menopausal women, but no significant association with skin viscoelastic properties was observed, suggesting that microbiome profiles are attributed to menopausal status rather than biophysical skin properties during aging. Dissecting microbial diversity by skin site, authors revealed differential microbiome diversity depending on skin site (cheek and forehead) and menopausal status, highlighting the menopause-associated physiological parameters as drivers that define skin microbiomes, opening the potential to exploit this knowledge for modulation of microbiomes in the management of skin disorders.

Lastly, Nurkolis et al. performed a complete revision exploring the salivary and skin microbiomes as detectors of aging-related diseases, supporting the aim of this topic to exploit information from microbiomes different from the gut. In this revision, authors explored numerous studies supporting that, despite skin and salivary microbiome are highly variable among individuals, specific changes in oral and skin microbiomes are linked to neurological, metabolic, skeletal, pulmonary, prostatic and macular diseases, and cancer, underpinning their potential for early detection and personalized treatment without invasive procedures.

In conclusion, novel insights regarding molecular mechanisms on gut microbiome and implications of skin and salivary microbiomes expand our knowledge into the functional role and application for the discovery of novel biomarkers. Understanding salivary and skin microbiomes opens up novel avenues for developing methods for the prevention, diagnosis, monitoring and treatment of aging-related diseases. Nonetheless, significant efforts are needed to validate candidate biomarkers, and a more exhaustive microbiome characterization is recommended, in special for some tissues such as skin, in which conclusions may differ depending on sample location.

